# A randomized, clinical trial to assess the relative efficacy and tolerability of two doses of etoricoxib versus naproxen in patients with ankylosing spondylitis

**DOI:** 10.1186/s12891-016-1275-5

**Published:** 2016-10-13

**Authors:** Eva Balazcs, Joachim Sieper, Kara Bickham, Anish Mehta, Nancy Frontera, Paul Stryszak, Zoran Popmihajlov, Paul M. Peloso

**Affiliations:** 1 Department of Neuromusculoskeletal Rehabilitation, Dr. Bugyi István Kórház, Sima Ferenc, Szentes, Hungary; 2 Charité Universitätsmedizin Berlin, Campus Benjamin Franklin, Berlin, Germany; 3 Merck & Co., Inc, Kenilworth, NJ USA

**Keywords:** Ankylosing spondylitis, NSAIDs, Etoricoxib, Naproxen, Spinal pain

## Abstract

**Background:**

This study evaluated two doses of etoricoxib (60 and 90 mg) vs. naproxen 1000 mg in subjects with ankylosing spondylitis (AS).

**Methods:**

This was a 2-part, double-blind, active comparator-controlled non-inferiority study in subjects ≥18 years of age with AS. In Part I, subjects were randomized to naproxen 1000 mg; etoricoxib 60 mg, and 90 mg. In Part II, naproxen and etoricoxib 90 mg subjects continued on the same treatment; subjects on etoricoxib 60 mg either continued on 60 mg or escalated to 90 mg. Part I (6 weeks) assessed the efficacy of A) etoricoxib 60 mg vs. naproxen and B) 90 mg vs. naproxen according to the time-weighted average change from baseline in Spinal Pain Intensity (SPI; 0–100 mm VAS) (primary endpoint). The non-inferiority margin was set at 8 mm for SPI. In Part II (20 weeks) we evaluated the potential benefit of increasing from 60 to 90 mg (predefined minimum clinically important difference = 6 mm in SPI) for inadequate responders (<50 % improvement from baseline in SPI) on etoricoxib 60 mg in Part I.

**Results:**

In total, 1015 subjects were randomized to receive etoricoxib 60 mg (*N* = 702), etoricoxib 90 mg (*N* = 156), and naproxen 1000 mg (*N* = 157); 70.9 % were male and the mean age was 45.2 years. There were 919 subjects who completed Part I and all continued to Part II. In Part I, SPI change was non-inferior for both etoricoxib doses vs. naproxen. In both Part I and II, the incidence of adverse events (AEs), drug-related AEs, and serious adverse events (SAEs) were similar between the 3 treatment groups.

**Conclusion:**

Both doses of etoricoxib were non-inferior to naproxen. All treatments were well tolerated. Etoricoxib 60 and 90 mg effectively control pain in patients with AS, with 60 mg once daily as the lowest effective dose for most patients.

**Trial registration:**

Clinical Trials Registry # NCT01208207. Registered on 22 September 2010.

**Electronic supplementary material:**

The online version of this article (doi:10.1186/s12891-016-1275-5) contains supplementary material, which is available to authorized users.

## Background

Ankylosing spondylitis (AS) is a chronic inflammatory spinal disorder in which patients most frequently report spinal pain, with pain at night and pain upon awakening being major features [1]. The chronic pain experienced by patients with AS often leads to interference in daily life and reduces the ability of patients to work and engage in social activities, thus impacting psychological well-being in addition to their physical disability [2]. Pain in AS has been associated with fatigue, which can also affect quality of life [3].

Non-steroidal anti-inflammatory drugs (NSAIDs) are treatments that have been used to treat pain and inflammation for several decades. In AS, they are recommended as first-line therapies by the ASsessment in AS international working group, the European League Against Rheumatism (ASAS/EULAR), and by the American College of Rheumatology (ACR) [4–6]. More recently, biologic therapies have become more widely adopted as treatments for AS and are associated with significant improvements of inflammatory burden and functional ability. However, NSAIDs have also demonstrated important treatment effects for AS patients. A recent systematic review suggests that there is high quality evidence from multiple randomized controlled trials suggesting that NSAIDs and COX-2 selective inhibitors are effective in managing spinal pain, peripheral joint pain and improving function [7]. In addition, NSAIDs have been shown to have additional therapeutic benefit in AS patients. NSAIDs inhibit prostaglandin synthesis through inhibition of the enzyme cyclooxygenase (COX). Prostaglandin E2 (PGE2) promotes osteoclast activity and stimulates osteoblast activity and in AS patients, this can potentially cause new pathologic bone formation. Previous studies have demonstrated that NSAIDs can slow radiographic progression in AS patients and may be more pronounced with COX-2 selective NSAIDs, although such findings could not be confirmed in all studies [8–10].

Etoricoxib, a COX-2 selective NSAID has demonstrated therapeutic benefit in patients with AS, as well as other acute and chronic pain conditions [11–16]. Results of a 52-week study in AS patients demonstrated that etoricoxib 90 mg and 120 mg were superior to placebo over 6-weeks and were superior to naproxen 500 mg BID (twice daily) over 1 year of treatment [16]. The efficacy of etoricoxib 90 and 120 mg did not differ, and thus, etoricoxib 90 mg was determined to be the most appropriate dose for AS patients.

This study was designed to further assess the dose-related efficacy and safety/tolerability profile of etoricoxib in subjects with AS by comparing etoricoxib 60 mg with naproxen 1000 and 90 mg with naproxen 1000 mg over 6 weeks and evaluating the potential benefit of increasing the etoricoxib dose to 90 mg in those subjects with inadequate pain responses to 60 mg.

## Methods

This study (Clinical Trials Registry # NCT01208207, Sponsor Protocol MK-0663 Protocol 108) was conducted at 187 study centers in Argentina, Austria, Belgium, Canada, Columbia, Czech Republic, Estonia, Finland, France, Germany, Hungary, India, Lithuania, Mexico, Poland, Romania, Russia, Slovakia, South Africa, Taiwan, the United Kingdom, and the United States. The study was initiated in September 2010 and completed in November 2014. The protocol for the study was approved by local institutional review boards or ethical review committees and was conducted according to principles of Good Clinical Practice. Subjects provided informed consent prior to participation in the study.

### Subjects

Subjects who were included were male or female, at least 18 years of age, and had a diagnosis of AS by the Modified New York criteria (determined/assessed at Visit 1) [17]. Subjects were to have had a history of positive therapeutic benefit with NSAIDs and were to have taken an NSAID on a regular basis and at a therapeutic dose level for at least 30 days prior to study enrollment. If subjects were using anti-rheumatic therapies other than NSAIDs, these were to be held at stable doses throughout the study duration. Bath Ankylosing Spondylitis Disease Activity Index (BASDAI) questionnaires were completed at each treatment visit and consisted of 6 questions pertaining to fatigue, spinal pain, joint pain/swelling, areas of localized tenderness, morning stiffness duration and morning stiffness severity. Subjects were required to have a baseline score on BASDAI Question 2 (Spinal Pain Intensity [SPI]; 0–100 mm on a visual analog scale [VAS]) at Visit 1 that was <77 mm. After “washout” of prestudy NSAID, subjects had to satisfy flare criteria before randomization. This flare criteria (i.e., worsening of spinal pain) was defined as a score on SPI after withdrawal of baseline NSAIDs that was: ≥40 mm, AND ≥30 % higher than the SPI score prior to NSAID withdrawal, AND ≥12 mm higher than the SPI prior to NSAID withdrawal.

Subjects were excluded from the study if they had conditions that would confound study results including a history of gastrointestinal (GI) surgery that causes clinical malabsorption, neoplastic disease, or a bleeding disorder (inherited or acquired). Additional conditions leading to exclusion were active peptic (gastric or duodenal) ulcer or history of inflammatory bowel disease, ischemic heart disease, cerebrovascular disease, or peripheral artery occlusive disease, class II-IV congestive heart failure, uncontrolled hypertension at Visit 1 or 2, BMI ≥40 kg/m^2^ and significant health problems stemming from obesity, estimated glomerular filtration rate ≤30 mL/min, or hepatic insufficiency (Child-Pugh score ≥5).

### Study design

This was a 2-part, double-blind, active-comparator-controlled study in subjects with AS. Part I was a 6-week, active-comparator-controlled period to compare the efficacy of etoricoxib 90 mg to naproxen1000 mg, the efficacy of etoricoxib 60 mg to naproxen 1000 mg and the efficacy of the 60 and 90 mg doses to each other. Part II was a 20-week, long-term treatment period that evaluated subjects who remained on naproxen 1000 mg, etoricoxib 90 mg, and etoricoxib 60 mg as well as subjects who escalated from etoricoxib 60 to 90 mg. Subjects were randomized at baseline (Visit 2) to one of four treatment sequences (treatment in Part I/treatment in Part II) in a 4:9:9:4 ratio: 1) naproxen 1000 mg/naproxen 1000 mg; 2) Etoricoxib 60 mg/etoricoxib 60 mg; 3) Etoricoxib 60 mg/etoricoxib 90 mg; 4) Etoricoxib 90 mg/etoricoxib 90 mg. Randomization was stratified by the presence or absence of chronic peripheral arthritis. Subjects were evaluated at Weeks 2, 4 and 6 in Part I. Subjects were evaluated at Weeks 10, 12, 18, and 26 in Part II.

Safety was monitored from screening throughout the study, and up to 28 days after the last dose of study medication.

### Efficacy parameters

The primary endpoint in this study was the time-weighted average change from baseline in SPI (100-mm VAS) over 6 weeks. The primary hypotheses were that etoricoxib 90 mg once daily is not inferior to naproxen 1000 mg according to the primary endpoint and that etoricoxib 60 mg once daily is not inferior to naproxen 1000 mg according to the primary endpoint. The Per Protocol Population (excludes subjects with important protocol deviations) was used for the analysis of the Primary Endpoint. Key Secondary Endpoints included time-weighted average change from baseline in SPI (VAS) in Part I (etoricoxib 90 mg vs. etoricoxib 60 mg; using the Modified Intention to Treat population [mITT]) and average change from Week 6 in SPI (VAS) over the average of Weeks 10 and 12 among inadequate responders to etoricoxib 60 mg (inadequate responder defined as a subject with <50 % improvement from baseline in SPI [VAS] at Week 6). Other Secondary Endpoints included time-weighted average change from baseline in SPI (VAS) over 26 weeks, time-weighted average response in Patient Global Assessment of Response to Therapy (PGART; Likert) over 6 weeks, proportion of subjects who discontinue due to lack of efficacy over 6 weeks, average change from Week 6 in PGART (Likert) over Weeks 10 and 12, and change from baseline in SPI (VAS) at each week of the study. We also evaluated the BASDAI composite score (VAS) and the morning stiffness duration and severity.

### Safety parameters

Safety assessments included physical examinations, vital signs, limited hematology and chemistry (i.e., hemoglobin, hematocrit, alanine aminotransferase [ALT], aspartate transaminase [AST], creatinine, and serum digoxin for subjects on digoxin), hematology [hemoglobin and hematocrit], urinalysis, serum or urine pregnancy testing, and serum FSH. Spontaneous adverse experience (AE) were monitored and reported up to 28 days (+2 days) after the last dose of study medication. All thrombotic CV SAEs and upper GI events (such as perforation, ulcer, or upper GI bleeding) that occurred in subjects in this study were subject to adjudication by a committee external to the Sponsor during the study.

### Statistical planning and analysis

The primary population analyzed for the primary hypothesis was the per-protocol population. The mITT population (randomized subjects who received at least 1 dose of study medication, had at least 1 post-randomization measurement for the analysis endpoint, and had baseline data) was used for a supportive analysis of the primary efficacy analyses and was the primary analysis population for all the secondary objectives. For evaluation of the primary objectives, the sample sizes in this study provided 91 % power (α = 0.025, 1-sided) for etoricoxib 90 mg vs. naproxen, >99 % (α =0.025, 1-sided) for etoricoxib 60 mg vs. naproxen. For secondary objectives, this study had 91 % power (α =0.20, 2-sided), and 80 % power (α =0.20, 2-sided) for etoricoxib 60 mg/90 mg vs. etoricoxib 60 mg/60 mg in inadequate responders.

The primary endpoint was analyzed using an analysis of covariance (ANCOVA). For the comparison of etoricoxib 90 mg vs. naproxen and the comparison of etoricoxib 60 mg vs. naproxen, non-inferiority was established if the upper bound of the 2-sided 95 % confidence interval of the between-treatment difference in the Least Square (LS) means (etoricoxib minus naproxen 1000 mg) was no larger than 8 mm VAS (non-inferiority margin based on a previous etoricoxib study in ankylosing spondylitis [16]). Etoricoxib 90 mg was declared superior to etoricoxib 60 mg if the difference in LS means for the primary endpoint was significant (*p* ≤ 0.20) in favor of the 90-mg dose. A difference of 6 mm VAS in the time-weighted average change from baseline over 6 weeks between two doses on SPI (VAS) is considered to be a Minimum Clinically Important Difference (MCID; i.e., a clinically important difference in means). This value was assigned after taking into account advice from consulted experts and data from dose range-finding studies since no specific literature was identified to aid in the assignment of MCID between doses in an AS population.

The comparison of inadequate responders in Part II was analyzed using ANCOVA. A difference of 6 mm VAS, as measured by the average change from Week 6 to Weeks 10 and 12 in SPI, was also assigned as a MCID for the comparison of etoricoxib 60 mg/etoricoxib 90 mg and etoricoxib 60 mg/etoricoxib 60 mg treatment sequences. The benefit of a dose increase from 60 to 90 mg was indicated if the nominal *p*-value (without multiplicity adjustment) for difference in LS means between the 2 treatment sequences was ≤0.20 in favor of the 90-mg dose.

The All Patients as Treated (APaT) population was used for the analysis of safety data (i.e., all randomized subjects who received at least 1 dose of study treatment). For pre-specified AEs of interest, *p*-values and/or 95 % CI’s were provided using the Miettinen and Nurminen method for treatment group comparisons. All other AEs were summarized with counts and percentages.

## Results

### Subjects

**Fig. 1:**
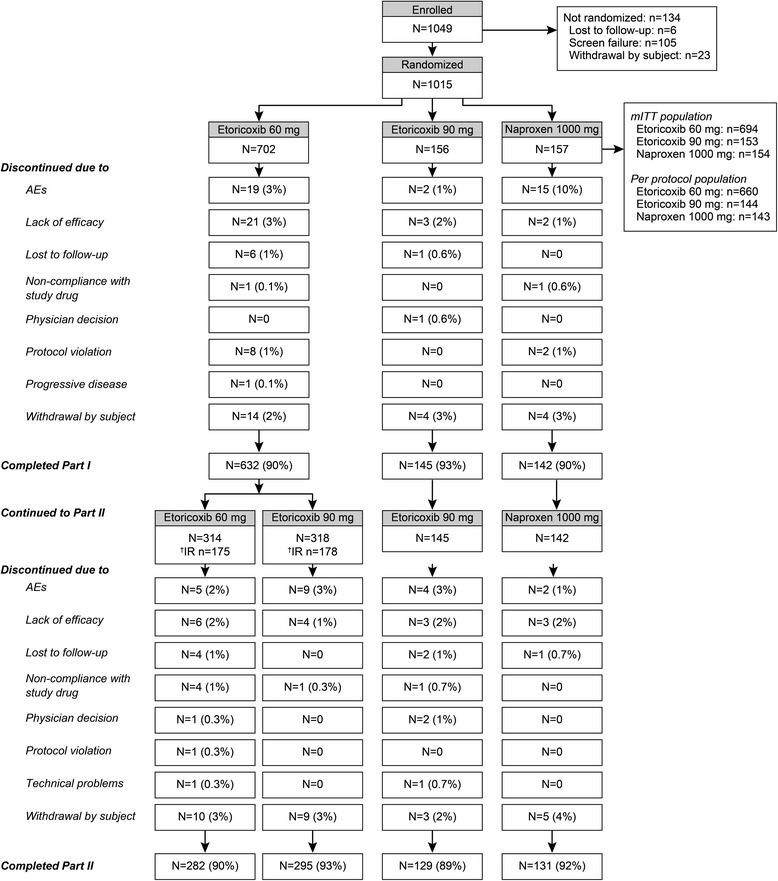
CONSORT Diagram/Study Design

There were 1015 subjects randomized to etoricoxib 60 mg (*n* = 702), etoricoxib 90 mg (*n* = 156), and naproxen 1000 mg (*n* = 157) in Part I. Of these randomized subjects, 919 subjects completed Part I (91 %). The most common reasons for early discontinuation were adverse events and lack of efficacy. Rates of discontinuation were low and similar across all treatment groups (Fig. 1). Of the 919 subjects entering Part II, 314 subjects continued receiving etoricoxib 60 mg, 145 subjects continued receiving etoricoxib 90 mg, 142 subjects continued receiving naproxen 1000 mg, and 318 subjects switched from etoricoxib 60 mg to etoricoxib 90 mg. There were 837 subjects who completed Part II (91 %), with similar distributions in the reasons for discontinuation between the treatment groups (Fig. 1). Baseline demographics were similar among the treatment groups; the majority of randomized subjects were male (70.9 %), subject ages varied from 19 to 82 years at enrollment, and the mean subject age at enrollment was 45.2 years. With regard to baseline disease characteristics in Part I, 302 (29.8 %) subjects had chronic peripheral arthritis, 244 (24.0 %) subjects had grade 4 bilateral sacroiliitis radiographic assessment according to the modified NY criteria. There were no clinically meaningful differences between the treatment groups for these or any other baseline disease-related characteristics (Table 1).

**Table 1:** Baseline Demographics

	Etoricoxib 60 mg	Etoricoxib 90 mg	Naproxen 1000 mg	Total
	*N* = 702	*N* = 156	*N* = 157	*N* = 1015
	n (%)	n (%)	n (%)	n (%)
Gender				
Female	209 (29.8)	45 (28.8)	41 (26.1)	295 (29.1)
Male	493 (70.2)	111 (71.2)	116 (73.9)	720 (70.9)
Age (Years)				
Mean (SD)	45.4 (12.4)	45.2 (11.3)	44.5 (12.3)	45.2 (12.2)
Range	19–82	19–82	21–75	19–82
Race				
White	589 (83.9)	132 (84.6)	138 (87.9)	859 (84.6)
Asian	88 (12.5)	17 (10.9)	16 (10.2)	121 (11.9)
Multi-racial	18 (2.6)	5 (3.2)	3 (1.9)	26 (2.6)
Black	4 (0.6)	2 (1.3)	0 (0.0)	6 (0.6)
Other	3 (0.4)	0 (0.0)	0 (0.0)	3 (0.3)
Chronic Peripheral Arthritis	213 (30.3)	44 (28.2)	45 (28.7)	302 (29.8)
Radiographic Assessment (bilateral grade 4) ^a^	177 (25.2)	38 (24.4)	29 (18.5)	244 (24.0)
BASDAI >4 at Screening n/N (%) ^b^	314/624 (50.3)	67/140 (47.9)	76/141 (53.9)	457/905 (50.5)
Mean (SD) Baseline Spinal Pain Intensity (0–100 mm VAS)	76.7 (14.2)	76 (15.2)	77.0 (14.0)	76.8 (14.3)

*SD* standard deviation, *VAS* visual analog scale

^a^ Radiographic assessment of bilateral sacroiliitis according to the modified NY criteria for AS

^b^ Responses to BASDAI questions were provided using a 0–100 mm Visual Analog Scale; the composite BASDAI score was divided by 10 to identify patients with BASDAI >4

### Efficacy

**Fig. 2:**
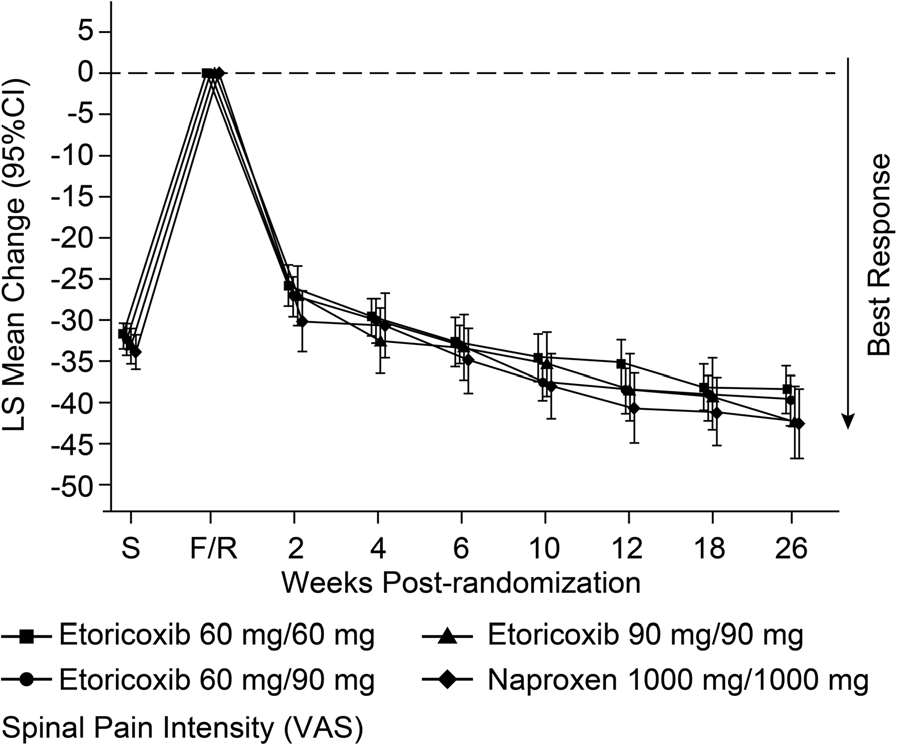
Spinal Pain Intensity over 26 Weeks

**Fig. 3:**
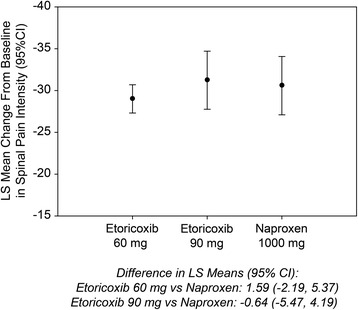
Time-weighted LS Mean Change from Baseline in Spinal Pain Intensity (0–100 mm VAS; Primary Endpoint, Part I)

For the primary endpoint of the time-weighted average change from baseline SPI score over 6 weeks of treatment in Part I, results were similar between the etoricoxib 60 mg, etoricoxib 90 mg, and naproxen 1000 mg groups (Figs. 2 and 3). For the comparisons of etoricoxib 60 mg vs. naproxen and 90 mg vs. naproxen, the upper limit of the 95 % CI was less than the pre-specified non-inferiority margin of 8 mm, thus etoricoxib 60 and 90 mg each demonstrated non-inferiority but not superiority to naproxen 1000 mg (Table 2).

**Table 2:** Summary of Primary and Secondary Efficacy Endpoints

	Naproxen 1000 mg	Etoricoxib 60 mg	LS mean difference vs. naproxen	Etoricoxib 90 mg	LS mean difference vs. naproxen	LS mean difference between 60 mg and 90 mg; *p*-Value
Primary Endpoint	*n* = 143	*n* = 660		*n* = 144		
Time-weighted LS Mean Change from Baseline in Spinal Pain Intensity (95 % CI) (PP population)	−30.59 (−34.07, −27.10)	−29.00 (−30.69, −27.31)	1.59 (−2.19, 5.37)	−31.23 (−34.70, −27.76)	−0.64 (−5.47, 4.19)	--
Secondary and Tertiary Endpoints	*n* = 154	*n* = 694		*n* = 154		
Time-weighted LS Mean Change from Baseline in Spinal Pain Intensity (95 % CI) (mITT population)	−30.84 (−34.19, −27.50)	−28.94 (−30.58, −27.29)	1.91 (−1.73, 5.55)	−30.51 (−33.87, −27.15)	0.33 (−4.33, 4.99)	−1.58 (−3.96, 0.81); *p* = 0.396
Time-weighted Average (LS Mean) Response over 6 Weeks in PGART (95 % CI) (mITT population)	2.19 (2.05, 2.32)	2.22 (2.16, 2.29)	0.03 (−0.11, 0.18)	2.29 (2.15, 2.42)	0.10 (−0.09, 0.29)	*N/A*
Proportion of Subjects Who Discontinued due to Lack of Efficacy (%)	2/154 (1.30)	20/694 (2.88)	1.58 (−0.60, 3.76)	2/154 (1.30)	0.65 (−2.17, 3.47)	*N/A*
Time-weighted LS Mean Change from Baseline in Bath Ankylosing Spondylitis Disease Activity Index (0–100 mm VAS)	−19.09 (−21.76, −16.42)	−19.34 (−20.65, −18.02)	−0.25 (−3.15, 2.66)	−20.60 (−23.28, −17.92)	−1.51 (−5.23, 2.21)	*N/A*
Time-weighted LS Mean Change from Baseline in Duration of Morning Stiffness (95 % CI) (0- to 100-mm VAS)	−15.13 (−17.98, −12.27)	−16.24 (−17.65, −14.83)	1.11 (−4.22, 2.00)	−18.29 (−21.16, −15.42)	−3.16 (−7.15, 0.82)	−2.05 (−5.17, 1.07)
Time-weighted LS Mean Change from Baseline in Level of Morning Stiffness over 6 weeks of Treatment (95 % CI) (0- to 100-mm VAS)	−19.35 (−22.49, −16.20)	−20.20 (−21.75, −18.64)	−0.85 (−4.27, 2.58)	−22.72 (−25.89, −19.56)	−3.38 (−7.76, 1.01)	−2.53 (−5.96, 0.91)
Time-weighted LS Mean Change from Baseline in Spinal Pain Intensity (95%CI) (PP population) for comparison of etoricoxib 60 mg vs. etoricoxib 90 mg	--	--	--	--	--	−2.23 (−4.69, 0.23); *p* = 0.246

*LS* least squares, *PP* per protocol, *mITT* modified intention to treat, *VAS* visual analog scale, *CI* confidence interval

For the comparison of etoricoxib 90 mg vs. 60 mg, the time-weighted average change from baseline SPI score was numerically greater for the etoricoxib 90 mg group compared to the etoricoxib 60 mg group over 6 weeks of treatment in Part I, but this difference did not achieve statistical significance at the pre-specified critical alpha = 0.20 (*p* = 0.396). In addition, the observed difference of 1.58 mm of the etoricoxib 90 mg dose over the etoricoxib 60 mg dose was less than the pre-specified MCID of 6 mm (Table 2). The time-weighted average response PGART score was similar between the etoricoxib 60 mg, etoricoxib 90 mg, and naproxen 1000 mg groups over 6 weeks of treatment in Part I (Table 2).

**Fig. 4:**
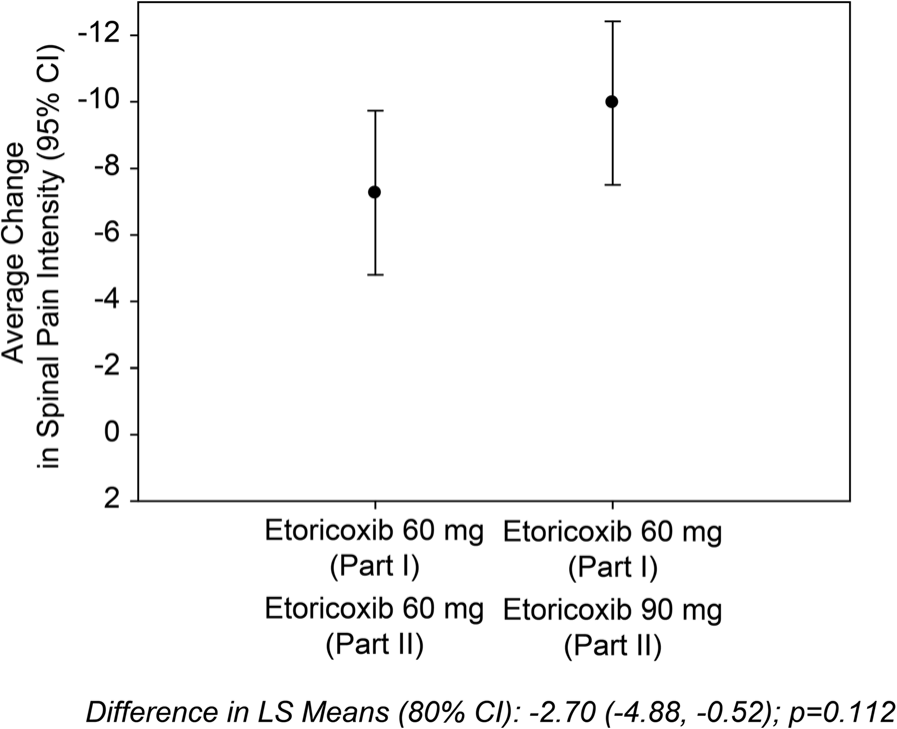
Average Change From Week 6 Over Weeks 10 and 12 (Part II) in Spinal Pain Intensity (0–100 mm VAS)

Additional secondary endpoints in this trial evaluated treatment effects in Part II of inadequate responders from Part I. In those subjects who were considered inadequate responders to etoricoxib 60 mg in Part I, the average change from Week 6 over the average of Weeks 10 and 12 in SPI score was statistically significantly better (at critical alpha = 0.20) in the group of inadequate responders who switched to etoricoxib 90 mg (*n* = 178) versus the group of inadequate responders who remained on etoricoxib 60 mg (*n* = 175) (*p* = 0.112), although the 2.70 mm difference observed was less than the prespecified MCID of 6 mm (Fig. 4, Table 3). The time-weighted average change from baseline SPI score was similar between the etoricoxib 60 mg/60 mg, etoricoxib 90 mg/90 mg, and naproxen 1000 mg/1000 mg groups over 26 weeks of treatment in the study (Table 3).

**Table 3:** Summary of Secondary Endpoints during Part II

	Naproxen 1000 mg (Part I)/1000 mg (Part II)	Etoricoxib 60 mg (Part I)/60 mg (Part II)	Etoricoxib 60 mg (Part I)/90 mg (Part II)	LS mean difference between etoricoxib 60 mg/90 mg vs. Etoricoxib 60 mg/60 mg; *p*-Value	Etoricoxib 90 mg (Part I/90 mg (Part II)
Number of Subjects	*n* = 74	*n* = 175	*n* = 178		*n* = 70
LS Mean Change from Week 6 in Spinal Pain Intensity Over Weeks 10 and 12 (95 % CI) (Inadequate Responders in Part I)	−12.89 (−16.57, −9.20)	−7.26 (−9.73, −4.80)	−9.97 (−12.42, −7.51)	−2.70 (−4.88, −0.52)	−7.69 (−11.48, −3.90)
LS Mean Change from Week 6 in PGART Over Weeks 10 and 12 (95 % CI) (Inadequate Responders in Part I)	0.21 (0.07, 0.35)	0.13 (0.04, 0.23)	0.21 (0.12, 0.30)	0.08 (−0.05, 0.21)	0.28 (0.13, 0.42)
Number of Subjects	*n* = 141	*n* = 310	*n* = 314		*n* = 140
Time-weighted LS Mean Change from Baseline in Spinal Pain Intensity Over 26 Weeks (95 % CI)	−38.26 (−41.70, −34.83)	−35.07 (−37.41, −32.73)	−36.30 (−38.65, −33.96)	−1.70 (−5.79, 2.39)	−36.76 (−40.22, −33.31)

*LS* least squares, *CI* confidence interval, *PGART* patient global assessment of response to therapy

The average change from Week 6 over Weeks 10 and 12 in PGART score was similar between the group of inadequate responders who remained on etoricoxib 60 mg and the group of inadequate responders who switched to etoricoxib 90 mg (nominal *p* = 0.221) (Table 3). The etoricoxib 60 mg and etoricoxib 90 mg groups had slightly higher proportions of subjects who discontinued due to lack of efficacy compared to the naproxen 1000 mg group; however, the nominal *p*-values for differences were only *p* = 0.401 and *p* > 0.999, respectively.

For the measure of time-weighted LS Mean change from baseline in the BASDAI score, etoricoxib 90 mg had the greatest change from baseline by the end of Part I, but the nominal *p*-value for the 90 mg dose vs. naproxen was 0.425; nominal *p*-value for the 60 mg dose vs. naproxen was 0.866. Results for the severity and duration of morning stiffness demonstrated a stronger numerical dose-related trend; however, nominal *p*-values for etoricoxib 90 mg vs. naproxen were 0.119 for duration and 0.131 for severity and for etoricoxib 60 mg vs. naproxen, nominal *p*-values were 0.483 for duration and 0.627 for severity (Table 3).

### Safety and tolerability

In Part I, 305 (30 %) subjects experienced an AE with a similar proportion in each treatment group. The most common AEs during Part I were upper abdominal pain, headache, and hypertension (Table 4). In Part II, 282 (31 %) subjects experienced an AE with a similar proportion of subjects in each treatment group. The most common AEs during Part II were nasopharyngitis and headache (Table 5). Among subjects who had SAEs, there was no dose-dependent trend during Part I. In Part II, a numerically higher proportion of subjects on the 90 mg dose or those who escalated to 90 mg from 60 mg had serious AEs compared with subjects who were on etoricoxib 60 mg throughout the study or for those in the naproxen group.

**Table 4:** Summary of AEs Part I

	Etoricoxib 60 mg *n* = 702 n (%)	Etoricoxib 90 mg *n* = 155 n (%)	Naproxen 1000 mg *n* = 156 n (%)
N (%) with AEs	222 (31.6)	36 (23.2)	47 (30.1)
N (%) with AEs determined by the investigator to be drug related	85 (12.1)	11 (7.1)	21 (13.5)
N (%) with serious AEs	5 (0.7)	1 (0.6)	0 (0.0)
N (%) who discontinued due to AEs	22 (3.1)	2 (1.3)	6 (3.8)
Most Common AEs (incidence >2 % in one or more treatment groups)			
Upper abdominal pain	19 (2.7)	2 (1.3)	6 (3.8)
Diarrhea	16 (2.3)	2 (1.3)	3 (1.9)
Nasopharyngitis	20 (2.8)	3 (1.9)	1 (0.6)
Dysgeusia	0 (0.0)	4 (2.6)	1 (0.6)
Headache	18 (2.6)	6 (3.9)	2 (1.3)
Hypertension	21 (3.0)	5 (3.2)	6 (3.8)
Serious AEs			
Appendicitis	0 (0.0)	1 (0.6)	0 (0.0)
Hip Fracture	1 (0.1)	0 (0.0)	0 (0.0)
Ankylosing Spondylitis	1 (0.1)	0 (0.0)	0 (0.0)
Cerebral Infarction	1 (0.1)	0 (0.0)	0 (0.0)
Cerebrovascular Accident	1 (0.1)	0 (0.0)	0 (0.0)
Headache	1 (0.1)	0 (0.0)	0 (0.0)
Prostatitis	1 (0.1)	0 (0.0)	0 (0.0)
Hypertensive crisis	1 (0.1)	0 (0.0)	0 (0.0)
Prespecified AEs of Interest			
Hypertension-related AEs	35 (5.0)	6 (3.9)	6 (3.8)
Discontinuation due to hypertension-related AEs	0 (0.0)	0 (0.0)	0 (0.0)
Edema-related AEs	4 (0.6)	0 (0.0)	0 (0.0)
Discontinuation due to edema-related AEs	0 (0.0)	0 (0.0)	0 (0.0)
Congestive heart failure, pulmonary edema, or cardiac failure	0 (0.0)	0 (0.0)	0 (0.0)

*AE* adverse event

**Table 5:** Summary of AEs Part II

	Etoricoxib 60 mg/60 mg *n* = 313 n (%)	Etoricoxib 60 mg/90 mg *n* = 319 n (%)	Etoricoxib 90 mg/90 mg *n* = 145 n (%)	Naproxen 1000 mg/1000 mg *n* = 142 n (%)
N (%) with AEs	97 (31.0)	103 (32.3)	38 (26.2)	44 (31.0)
N (%) with AEs determined by the investigator to be drug related	20 (6.4)	20 (6.3)	7 (4.8)	12 (8.5)
N (%) with serious AEs	1 (0.3)	7 (2.2)	5 (3.4)	2 (1.4)
N (%) who discontinued due to AEs	3 (1.0)	9 (2.8)	4 (2.8)	2 (1.4)
Serious AEs				
Angina Pectoris	0 (0.0)	1 (0.3)	0 (0.0)	0 (0.0)
Left ventricular hypertrophy	0 (0.0)	1 (0.3)	0 (0.0)	0 (0.0)
Glaucoma	1 (0.3)	0 (0.0)	0 (0.0)	0 (0.0)
Gastric Ulcer	0 (0.0)	1 (0.3)	0 (0.0)	0 (0.0)
Gastric Ulcer Hemorrhage	0 (0.0)	1 (0.3)	0 (0.0)	0 (0.0)
Death	0 (0.0)	1 (0.3)	0 (0.0)	0 (0.0)
Non-cardiac Chest Pain	0 (0.0)	0 (0.0)	0 (0.0)	1 (0.7)
Abscess	0 (0.0)	0 (0.0)	0 (0.0)	1 (0.7)
Diverticulitis	0 (0.0)	1 (0.3)	0 (0.0)	0 (0.0)
Sialoadenitis	0 (0.0)	0 (0.0)	0 (0.0)	1 (0.7)
Contusion	0 (0.0)	0 (0.0)	1 (0.7)	0 (0.0)
Rib Fracture	0 (0.0)	0 (0.0)	1 (0.7)	0 (0.0)
Skin Abrasion	0 (0.0)	0 (0.0)	1 (0.7)	0 (0.0)
Ankylosing Spondylitis	0 (0.0)	1 (0.3)	0 (0.0)	0 (0.0)
Rotator Cuff Syndrome	0 (0.0)	0 (0.0)	1 (0.7)	0 (0.0)
Ear Neoplasm	0 (0.0)	1 (0.3)	0 (0.0)	0 (0.0)
Renal Cell Carcinoma	0 (0.0)	1 (0.3)	0 (0.0)	0 (0.0)
Ischemic Stroke	0 (0.0)	0 (0.0)	1 (0.7)	0 (0.0)
Depression	0 (0.0)	1 (0.3)	0 (0.0)	0 (0.0)
Pulmonary Embolism	0 (0.0)	0 (0.0)	2 (1.4)	0 (0.0)
Deep Vein Thrombosis	0 (0.0)	0 (0.0)	1 (0.7)	0 (0.0)
Hypertension	0 (0.0)	1 (0.3)	0 (0.0)	0 (0.0)
Most Common AEs (incidence >2 % in one or more treatment groups)				
Upper Abdominal Pain	7 (2.2)	2 (0.6)	0 (0.0)	4 (2.8)
Influenza	1 (0.3)	2 (0.6)	2 (1.4)	3 (2.1)
Nasopharyngitis	9 (2.9)	11 (3.4)	4 (2.8)	3 (2.1)
Urinary tract infection	2 (0.6)	7 (2.2)	0 (0.0)	1 (0.7)
Contusion	2 (0.6)	0 (0.0)	3 (2.1)	3 (2.1)
Arthralgia	8 (2.6)	2 (0.6)	2 (1.4)	0 (0.0)
Headache	6 (1.9)	7 (2.2)	5 (3.4)	4 (2.8)
Hypertension	9 (2.9)	9 (2.8)	3 (2.1)	4 (2.8)
Prespecified AEs of Interest				
Hypertension-related AEs	14 (4.5)	10 (3.1)	3 (2.1)	4 (2.8)
Discontinuation due to hypertension-related AEs	0 (0.0)	1 (0.3)	0 (0.0)	0 (0.0)
Edema-related AEs	4 (1.3)	4 (1.3)	0 (0.0)	3 (2.1)
Discontinuation due to edema-related AEs	0 (0.0)	0 (0.0)	0 (0.0)	0 (0.0)
Congestive heart failure, pulmonary edema, or cardiac failure	0 (0.0)	0 (0.0)	0 (0.0)	0 (0.0)

*AE* adverse event

With regard to prespecified AEs of interest, which included hypertension-related AEs, edema-related AEs, and AEs of congestive heart failure, pulmonary edema, or cardiac failure, there were no statistically significant differences between the treatment groups in either Part I or Part II; while naproxen had a numerically greater proportion of subjects with hypertension-related AEs, there were no other dose-dependent trends in the prespecified AEs of interest.

There were 2 subjects with a total of 3 confirmed/adjudicated GI events during the study (1 subject in the etoricoxib 60 mg/60 mg group and 1 subject [with 2 events] in the etoricoxib 60 mg/90 mg group during Part II). The events of gastric ulcer and gastric ulcer hemorrhage experienced by the subject in the etoricoxib 60 mg/90 mg group were confirmed by the adjudication committee as upper GI bleeds.

There were seven subjects experiencing 8 thrombotic CV AEs. Two subjects in the etoricoxib 60 mg group in Part I had an ischemic stroke. In Part II, 2 subjects in the etoricoxib 60 mg/90 mg group had confirmed thrombotic CV events (acute myocardial infarction and sudden death [cause unknown]); 2 subjects had 3 events in the 90 mg/90 mg group during Part II (one subject had an ischemic stroke, one subject had a pulmonary embolism, and one subject had a peripheral venous thrombosis and pulmonary embolism).

One death occurred in the etoricoxib 60 mg/90 mg group during the follow-up period after completion of treatment. The subject was not hospitalized prior to death, an autopsy was not conducted, and the cause was determined to be unknown; for these reasons, the adjudication committee could not rule out that the subject had a thrombotic CV event resulting in death.

## Discussion

In this study, both etoricoxib 90 and 60 mg were non-inferior to naproxen 1000 mg on the primary endpoint of time-weighted average change from baseline in the SPI score of a 6-week period. These results were further validated by other endpoints, including PGART and discontinuations due to lack of efficacy. An analysis was done for the secondary objective comparing the effect of etoricoxib 90 mg vs. etoricoxib 60 mg on the time-weighted average change from baseline SPI score; the difference in the effect did not meet the prespecified MCID. All other secondary and tertiary endpoints supported similar efficacy between the doses in Part I.

Among the subset of subjects who did not have an adequate response to the 60 mg dose during Part I, those who received the 90 mg dose in Part II demonstrated an additional average improvement of ~3 mm in SPI score from Week 6 over Weeks 10 and 12 as compared to subjects who continued receiving the 60 mg dose in Part II. This result was identified as being statistically significant in this study; however, the results were not supported by PGART results. The 3-mm average improvement with the 90 mg dose vs. the 60 mg dose also did not meet the predefined MCID of 6 mm. Further research evaluating MCID on an individual patient level rather than as an average measure may be useful in determining if the numerically greater improvement from the 90 mg dose provides clinically important effects for some AS patients. Overall, while etoricoxib previously demonstrated efficacy in subjects with AS at a dose of 90 mg [16], our results indicate that etoricoxib 60 mg is a clinically relevant dose that provides efficacy for a majority of patients similar to that achieved with 90 mg. An effective lower dose of medication may decrease a patient’s potential adverse effects, and treatment interruptions. In the Multinational Etoricoxib and Diclofenac Arthritis Long-term (MEDAL) study, which was a large outcome trial that was designed to evaluate safety parameters among osteoarthritis (OA) and rheumatoid arthritis patients who received etoricoxib 60 mg, etoricoxib 90 mg, and diclofenac 150 mg, the 60 mg dose (received by OA patients) was associated with fewer discontinuations due to AEs or serious AEs compared with OA patients who received etoricoxib 90 mg. Additionally, a lower rate of congestive heart failure and discontinuations due to edema were observed with etoricoxib 60 mg vs. etoricoxib 90 mg in the MEDAL trial [18, 19]. In the current study, all treatments were generally well tolerated with no new or unexpected safety findings. There were no significant differences in AEs between treatment groups; however a small numeric increase in SAEs was noted in Part II in 90 and 60 mg/90 mg treatment arms as compared to 60 mg treatment arm.

Due to the cardiovascular and gastrointestinal risks associated with NSAIDs [20], these events were adjudicated by an external committee of experts in this trial. Further, it should be noted that CV risk is elevated in AS patients [21]. The proportion of subjects who experienced thrombotic CV or upper GI events was relatively low and not unexpected in this study. The thrombotic CV events that occurred in this study were in the etoricoxib groups with none occurring in the naproxen group; however, the incidence was too low to adequately assess risk. Previous analyses have suggested that NSAIDs, including COX-2 selective NSAIDs such as etoricoxib, have a similar increased risk of CV events, although with the possible exception of naproxen which is not associated with an increased risk [20]. A large outcome program (the MEDAL program) demonstrated a similar rate of thrombotic CV events with etoricoxib (60 or 90 mg) and diclofenac 150 mg; the MEDAL program also demonstrated a reduced risk of uncomplicated upper GI events with etoricoxib vs. diclofenac [22].

This study demonstrated that etoricoxib 60 and 90 mg are clinically important doses in the treatment of AS and are non-inferior to naproxen 1000 mg with regard to reduction of spinal pain intensity. However, the limitation of this study was that assessments of the efficacy of these doses on an individual patient level were not studied. Additionally, these treatments were only assessed based on clinical endpoints. Previous research demonstrated radiographic improvement in AS patients treated with NSAIDs, COX-2 selective NSAIDs in particular, presumably due to inhibition of osteoblast activity [9]. Though not assessed in this study, the effect of etoricoxib and other COX2 inhibitors on radiographic disease progression in AS patients is a potentially important area for future research.

## Conclusions

In summary, both etoricoxib 90 and 60 mg were well tolerated in this study, and no new safety signals were identified. The sum of the evidence from this study suggests that etoricoxib 60 and 90 mg effectively control pain and a choice of two effective doses (60 mg or 90 mg) has now been described for patients with AS, with 60 mg once daily as the lowest effective dose for most patients. This choice of two effective doses provides healthcare providers with an additional option to optimize AS treatment based on individual patient response.

## Additional file

Additional file 1: List of Ethics Committees. (DOCX 27 kb)

## Abbreviations

AE: Adverse event
ALT: Alanine aminotransferase
ANCOVA: Analysis of covariance
APaT: All patients as treated
AS: Ankylosing spondylitis
ASAS: ASsessment in AS
AST: Asparate transaminase
BASDAI: Bath ankylosing spondylitis disease activity index
BID: Twice daily
BMI: Body mass index
COX: Cyclooxygenase
CV: Cardiovascular
EULAR: European league against rheumatism
GI: Gastrointestinal
MCID: Minimum clinically important difference
MEDAL: Multinational etoricoxib and diclofenac arthritis long-term
mITT: Modified intention to treat
NSAID: Non-steroidal anti-inflammatory drug
OA: Osteoarthritis
PGART: Patient global assessment of response to therapy
PGE2: Prostaglandin E2
SPI: Spinal pain intensity
VAS: Visual analog scale
