# Supplementary material for: A randomized, clinical trial to assess the relative efficacy and tolerability of two doses of etoricoxib versus naproxen in patients with ankylosing spondylitis
Source: BMC Musculoskelet Disord. 2016 Oct 13;17:426. doi: 10.1186/s12891-016-1275-5 (PMC5062857; doi:10.1186/s12891-016-1275-5)
Supplement: Additional file 1: — List of Ethics Committees. (DOCX 27 kb) [file 12891_2016_1275_MOESM1_ESM.docx]

**List of Ethics Committees**

Hospital Privado Centro Medico

Naciones Unidas 346

Vélez Sarsfield, Córdoba (X5016KEH)

Instituto CAICI SRL.

Mendoza 2612

Rosario, Santa Fe (S2000PBJ)

Organización Médica de Investigación (OMI)

Uruguay 725 PB, Ciudad Autónoma de Bs As (C1015ABO), Argentina / Tel +54 11

4372 0308

Comité Independiente de Ética para ensayos en farmacología Clínica

Av. J.E. Uriburu 774 1°Piso (C1027AAP), Buenos Aires

TE/FAX: 5411 4952-3892

Ethics Committee of the City of Vienna

1030 Vienna, Thomas-Klestil-Platz 8, Town Town 1st Floor, CB 12.103

Access: 1030 Vienna, Schnirchgasse 12, Staircase 2, CB 12.103

Ethikkommission des Landes Niederösterreich

Abteilung Sanitäts- und Krankenanstaltenrecht

Herr Mag. R. Bruckner. Landhausplatz 1Haus 15 b. A-3109 St. Pölten

Ethikkommission des Landes Oberösterreich

Landesnervenklinik Wagner-Jauregg

Herr Univ.-Prof. Prim. Dr. J. Fischer

Wagner-Jauregg Weg 15. A-4020 Linz

University of Liège Hospital-Faculty Ethics Committee

Sart Tilman University Domain – B35, 4000 LIEGE 1

ethique@chu.ulg.ac.be. Tel.: 04 366 83 10

Schulman Associates IRB

4290 Glendale-Milford Rd, Cincinnati, Ohio (45242)

Biomedical Research Ethics Committee médico Julián Coronel

Cra 59 N° 1E-21, Cali, Valle, Colombia. PBX:6800202

Fernando Chalem Rheumatology Institute Foundation

Calle 73 No. 20A - 27 - Te: 346 6077 Fax: 255 8381 Bogotá, D.C.

CEI Servimed E.U (Ethics committee)

Calle 51 N° 34-17 consultorios 208 C.C. Cabecera, Bucaramanga, Colombia. Te:

6432426. [ceiservimedeu@hotmail.com](mailto:ceiservimedeu@hotmail.com)

El Oriente Research Ethics Committee

Calle 53 # 34 - 20 – Cabecera, Bucaramanga – Colombia

Telephones: (57) 300 618 4784

The Research Ethics Committee - Riesgo de Fractura

Carrera 13 No.97- 25 - Bogotá, TEL: 6446800 - 5922991 ext. 502

Ethics Committee for Multi-Centric Clinical Trial of the University Hospital Motol

V úvalu 84, 150 06 Praha 5. Tel 224 431 195

Local Ethics Commitee Úrazové nemocnice v Brne

Ponavka 6. 662 50 Brno

tel:+420 545538666, fax: 545211082, e-mail [reditelstvi@unbr.cz](mailto:reditelstvi@unbr.cz)

Local Ethics Committee of the Hospital in Chomutov

Kochova 1185, 43012 Chomutov Krajská zdravotní a.s., Nemocnice Chomutov o.z.

Ethics Committee for Multi-Centric Clinical Trial of the University Hospital Motol

V úvalu 84, 150 06 Praha 5. Tel 224 431 195

Tallinn Medical Research Ethics Committee . Hiiu 42, Tallinn 11619, Estonia

+372 659 3924. marje.liibek@tai.ee. [www.tai.ee](http://www.tai.ee)

Comité de Protection des Personnes Ile de France VIII

Hôpital Ambroise Paré - 9, avenue Charles de Gaulle. 92100 Boulogne-Billancourt.

Phone: 01 49 09 58 14

Medisiininen eettinen toimikunta, HUS

Biomedicum Helsinki 2 C Tukholmankatu 8 C, 7 krs.

PL 705. 00029 HUS. Finland

Ethics Committee of the Medical Faculty of the University of Leipzig

Institute of Clinical Pharmacology, Härtelstraße 16-18, 04107 Leipzig

Ethics Committee for Clinical Pharmacology

H-1051 Budapest, Arany J.u. 6-8. Hungary. Ph: +36-1-795-1195.

Prof

IEC – Krishna Institute of Medical Sciences Ltd

#1-8-31/1, Minister Road, Secunderabad-500 003, A.P., India

+91 040 4488 5000. [Info@kims.com.in](mailto:Info@kims.com.in)

Swastic Independent Ethics Committee

B-130, Modi Nagar, Ajmer Road Jaipur- 302019. [swasticethics@gmail.com](mailto:swasticethics@gmail.com)

Bhagwan Mahavir Medical Research Centre

10-1-1, Bhagwen Mahavir Marg, A.C. Guards, Hyderabad – 500 004, A.P. India

Ethics Committee – Shalby Hospitals

Opp. Karnavati Club, Sarkej Gandhingar Highway, Ahmedabad 38001.

[info@shalby.org](mailto:info@shalby.org)

Hyderabad Central Ethics Committee

#12-13-392. Street N°1. Lane No 4. Tarnaka. Hyderabad-500017. Andhra Pradesesh,

India. [hyderabadcec@gmail.com](mailto:hyderabadcec@gmail.com)

Penta-Med Ethics Committee. C/O Medipoint Hospitals Pvt. Ltd

241/1 , New D.P. Road, Aundh. Pune 411 007. Phone: 020 3984 1206

Lithuanian Bioethics Committee

State budget institution, Didzioj Str. 22. LT-01128 Vilnius, Lithuania

ibek@sam.lt. (+370 5) 212 4565

Instituto Jalisciense de Investigación Clínica S.A de C.V.

Penitenciaria N° 20, Col. Centro, Guadalajara, Jalisco. CP 44100.

Ph. 38 25 64 93

Centro de Investigación Clínica Especializada – Research Ethics Committee

Tlatetilpa #24, Col. Barrio San Lucas, Mexico D.F., C.P. 04030.

Tel./Fax: 53364206 and 52507456. comite.cice@prodigy.net.mx

Bioethics Committee at Investigación Clínica S.A.

Puebla No. 422 -4, Col. Roma, 06700 Mexico D.F. Tel: 5256-4910

Ethics Committee Hospital de Jesús

Av. 20 de Noviembre 82, C.P. 06090 Mexico D.F.

Hospital Civil Fray Antonio Alcalde

Hospial N° 278 S.H. C.P. 44280, Guadalajara, Jalisco. Tel. 3614-5501

Komisja Bioetyczna przy Okręgowej

Izbie Lekarskiej w Białymstoku

(Bioethics Committee at the Regional Medical Chamber of Physicians and Dentists in

Białystok) Ul. Świetojańska 7. 15-082 Białystok

Komisja Bioetyczna przy Okręgowej

Izbie Lekarskiej w Gdańsku (Bioethics

Committee at the Regional Chamber of Physicians and Dentists in

Gdańsk) Ul. Śniadeckich 33. 80-204 Gdańsk

Bioethics Committee at the Regional Medical Council of Regional Chamber of

Physicians and Dentists in Poznań

61-734 Poznań, ul. Nowowiejskiego 51. Tel. (0-61) 852-58-60, Fax.: (061) 851-87-62

Bioethics Committee at the Regional Medical

Council of Regional Chamber of Physicians and Dentists in Poznań

61-734 Poznań, ul. Nowowiejskiego 51

Tel. (0-61) 852-58-60, Fax.: (061) 851-87-62

Komisja Bioetyczna przy Okręgowej Radzie Lekarskiej Wielkopolskiej Izby

Lekarskiej (Bioethics Committee at the Regional Medical Council of the Regional

Chamber of Physicians and Dentists in Poznań)

Ul. Nowowiejskiego 51. 61-734 Poznań

Bioethics Committee at the Regional Medical

Council of Regional Chamber of Physicians and Dentists in Poznań

61-734 Poznań, ul. Nowowiejskiego 51

Tel. (0-61) 852-58-60, Fax.: (061) 851-87-62

Komisja Bioetyczna przy Okręgowej

Izbie Lekarskiej w Krakowie (Bioethics Committee at the Regional

Chamber of Physicians and Dentists in Cracow)

Ul. Krupnicza 11a

31-123 Krak6w

Komisja Bioetyczna przy Okręgowej Radzie Lekarskiej Wielkopolskiej Izby

Lekarskiej (Bioethics Committee at the Regional Medical Council of the Regional

Chamber of Physicians and Dentists in Poznań)

Ul. Nowowiejskiego 51. 61-734 Poznań

Komisja Bioetyczna przy Okręgowej

Izbie Lekarskiej w Warszawie

(Bioethics Committee at the Regional

Chamber of Physicians and Dentists

in Warsaw) Ul. Puławska 1802-512 Warszawa

0409 Bioethics Committee at the Regional Medical

Council of Regional Chamber of Physicians and Dentists in Poznań

61-734 Poznań, ul. Nowowiejskiego 51

Tel. (0-61) 852-58-60, Fax.: (061) 851-87-62

Bioethics Committee at the Regional Medical

Council of Regional Chamber of Physicians and Dentists in Poznań

61-734 Poznań, ul. Nowowiejskiego 51

Tel. (0-61) 852-58-60, Fax.: (061) 851-87-62

Bioethics Committee at the Regional Medical

Council of Regional Chamber of Physicians and Dentists in Poznań

61-734 Poznań, ul. Nowowiejskiego 51

Tel. (0-61) 852-58-60, Fax.: (061) 851-87-62

Bioethics Committee at the Regional Medical

Council of Regional Chamber of Physicians and Dentists in Poznań

61-734 Poznań, ul. Nowowiejskiego 51

Tel. (0-61) 852-58-60, Fax.: (061) 851-87-62

Ministry of Health. National Ethics Committee for Clinical Study of Medicines.

011478 Bucharest, 48 Av. Sanatescu Street, district 1

Phone: 0314051076; Fax: 0314051075

State Budgetary Educational Institution of Higher Professional Education

KEMEROVO STATE MEDICAL ACADEMY FEDERAL SERVICE ON

SURVEILLANCE IN HEALTHCARE AND SOCIAL DEVELOPMENT

*22a Voroshylova ul., Kemerovo, 650029*

*tel. (8-3842) – 734856, fax (8-3842) – 734856*

GBKUZ of Yaroslavl region “Cit

7, Semashko ul. Yaroslavl Yaroslavskaya oblast' 150002 RUSSIAN FEDERATION /

Tel +79038240506

State Healthcare Institution Regional Clinical Hospital

1 Smirnovskoe Gorge, Saratov,Russia, 1410053

Tel.: +7 8452 491467

Independent Ethics Committee of Smolensk State Medical Academy

28 ul. Krupskoj, 214019, Smolensk, Russia

Rheumatology Consultation and Diagnostics Center “Healthy Joints”

33 Romanov St., Novosibirsk, Russia, 630091

Tel.: +7 (383) 33100 49; Fax: +7 (383) 222-54-03

e-mail: info@zsustav.ru; Web-site: [www.zsustav.ru](http://www.zsustav.ru)

KIMI-LEC

197110, St Petersburg, Krestovskiy pr., 18, tel. +7 921 930 9984

Ethics Committee at Gosudarstvennoye byudzhetnoye uchrezhdeniye

zdravookhraneniya [State Budgetary Healthcare Institution] of the Republic of

Karelia.

Medical Institute for Scientific Research «Your Health» LLC

2 Dostoyevskiy St., Kazan, Russia, 420097

Tel./Fax: (843) 537 93 93

State Healthcare Institution of the City of Moscow Municipal Clinical Hospital No. 1

n.a. N.I. Pirogov

8 Leninskiy Ave., Moscow, Russia, 119049

Tel.: (495)2366096, Fax: (495)2366528

Urad Trnavskeho Samospra Vneho Kraja

Eticka Komisia. P.O BOX 128, Strarohajska 10

917 01 Trnava

Ethics Committee of Bratislava Self-Governing Region

Sabinovska 16, 820 05 Bratislava

National Institute of Rheumatic Diseases

Nábr. I. Krasku 4, 921 12 Piešťany, Slovak Republic

Pharma-Ethics Independent Research Ethics Committee

123 Amcor Road, Lyttelton Manor, 0157

Institutional Review Board Chang Gung Medical Foundation

No. 199, Tung Hwa North Road, Taipei City 105 . Fax: 03-3494549

Contact Person & Phone: Ting-Yi Wu; 03-3196200 ext. 3704

Taipei Veterans General Hospital, Vac

201 Shih-pai road, Sec 2. Taipei, Taiwan. Republic of China

China Medical University Hospital IRB

No. 2 Yuh Der Road, Taichung Taiwan R.O.C

Institutional Review Board of Buddhist Dalin Tzu Chi General Hospital

2, Min-Sheng Road, Dalin Town, Chia-Yi. TAIWAN R.O.C (622)

Tel: 05-2648000

Yi-Gong Li

0223 Joint Institutional Review Board

No.5-1 Lane 331, Sec 2, Shih-Pai Road, Taipei (11217), Taiwan. R.O.C

Low-Tone HO

Institutional Review Board Committee, Changhua Christian Hospital

135, Nan-Hsiao Street, Changhua 500, Taiwan

Buddhist Tzu Chi General Hospital Research e Ethics Committee

707, Sec.3, Chung-Yang Rd., Hualien, 97002, Taiwan, R.O.C.

Institutional Review Board Chung Shan Medical University Hospital

No. 110, Sec.1, Chien-Kuo N. Road, Taichung, Taiwan 402, R.O.C

Research Ethics Committee National Taiwan University Hospital

7, Ching-Shan South Road, Taipei, Taiwan 100, R.O.C

National Research Ethics Services Cambridgeshire 2 Research Ethics Committee.

Victoria House, Capital Park, Fulbourn, Cambridge CB21 5XB

Schulman Associates IRB, Inc

4445 Lake Forest Drive Suite 300, Cincinnati Ohio 45242 UNITED STATES / Tel

888-557-2472 / [dbattson@sairb.com](mailto:dbattson@sairb.com)
